# Secular trends of methicillin-resistant Staphylococcus aureus (MRSA) at Geneva University Hospitals (HUG) over a 14-year period

**DOI:** 10.1186/2047-2994-4-S1-O9

**Published:** 2015-06-16

**Authors:** C Fankhauser, J Schrenzel, P Francois, D Pittet, S Harbarth

**Affiliations:** 1 Infection Control Programme, Geneva University Hospitals, Geneva, Switzerland; 2 Genomic Research Laboratory, Geneva University Hospitals, Geneva, Switzerland; 3 Bacteriology Laboratory, Geneva University Hospitals, Geneva, Switzerland

## Introduction

Controlling MRSA has been a challenge for Geneva University Hospitals (HUG), particularly after the introduction of ST228 SCCmecI hyperendemic clone in 1999.

## Objectives

To describe HUG’s secular trends of MRSA, infection control measures and predominant MRSA clones using Staphylococcal chromosomic cassettes (SCCmec) genotyping.

## Methods

A multifaceted MRSA prevention program initiated in 1993 included patient screening, decontamination, surveillance, contact isolation, an alert system and a hospital-wide hand hygiene campaign (HHC); subsequently strengthened by: an educational campaign (2003), routine MRSA SCCmec genotyping (2005), a 2^nd^ HHC, periodic audits and feedback (2006). The intensive care unit performed MRSA screening on admission, discharge and weekly since 2004. Surveillance included: (1) incidence rates (IR) of hospital acquired (HA)-MRSA infection or colonization; (2) HA-MRSA bloodstream infections (BSI); (3) proportion of MRSA/*S. aureus* BSI; (4) IR of MRSA clinical cultures (CC); (5) proportion of SCCmec in MRSA strains, assessed by routine multiplex PCR assay. Representative isolates were grouped in MLVA clusters to evaluate genomic diversity and subjected to MLST.

## Results

At HUG, from 2000-2014, 12,543 patients were MRSA-colonized or infected (incl. >75% screening swabs; 530 BSI). From 2000-2007, annual rates of all indicators showed an increasing trend, declining since 2008. New HA-MRSA cases per 100 admissions increased from 1.36 to 2.00 (2006), and then declined to 0.29 (2014). Trends expressed by incidence density of cases per 1000 hospital-days: HA-MRSA, from 0.92 to 1.36 (2007) to 0.21 (2014); ICU-acquired HA-MRSA from 2.3 (2002) to 10.5 (2006) to 1.31 (2014); MRSA-CC rates from 0.68 to 1.44 (2008), to 0.24 (2014); HA-BSI from 0.049 to 0.07 (2009), to 0.016 (2014). The proportion of MRSA/*S. aureus* BSI remained around 34% (2000-2009), declining to 18% (2014). SCCmecI strains declined from 83% (2005) to 32% (2014); SCCmecII and SCCmecIV were higher in non-acute settings.

## Conclusion

A multifaceted prevention program and possible changes in biologic fitness of the ST228 SCCmecI clone helped to decrease endemic MRSA rates for the last 7 years.

## Disclosure of interest

None declared.

